# Peer victimisation and drug use in sexual minorities

**DOI:** 10.1192/j.eurpsy.2025.966

**Published:** 2025-08-26

**Authors:** R. S. C. Lee, A. Curley

**Affiliations:** 1 Melbourne School of Psychological Sciences, The University of Melbourne, Melbourne, Australia

## Abstract

**Introduction:**

Adversity in adolescence, including peer victimisation, is associated with substance misuse in young adults, particularly in vulnerable individuals like sexual minorities. However, the potential developmental mechanisms underlying this association are yet to be fully understood.

**Objectives:**

This study will empirically investigate the relationship between childhood adversity and addictive behaviours in young adulthood (i.e., drug use). In particular, we will examine the possible moderating role of sexual identity and orientation on drug use problems.

**Methods:**

A total of 329 adults (aged 18 to 35 years old) were recruited into the study and included in the final analysis. Of the 329 participants, 93 identified as being a sexual minority (26.16%). A large majority of participants were women (N = 278 / 78.1%) with a mean age of 20.3 years old (SD = 3.5) and a diverse distribution of ethnicities reflective of metropolitan Australia. All participants completed an online battery of demographic, self-report, and behavioural measures. A multiple regression using Hayes’ PROCESS macro for SPSS was conducted.

**Results:**

Overall, both identifying as being a sexual minority (b = .51, p <. 05) and reporting greater peer victimisation during childhood (b = .17, p <. 01) predicted greater levels of drug use in adulthood. Over and above the independent effects of sexual orientation and peer victimisation, being a sexual minority who also experienced a high level of peer victimisation were together predictive of more pronounced drug use in adulthood (b = .25, p <. 05).

**Conclusions:**

Identifying as being a sexual minority as well as reporting greater peer victimisation in childhood were independently predictive of potential risky drug use in adulthood. This is in keeping with theories of the role of chronic stress in the development of potentially harmful behavioural, coping mechanisms. Consistent with our hypothesis, these effects were magnified when they occurred in combination, such that sexual minorities were more susceptible to the effects of peer victimisation on later drug use. Findings from the current study contribute to the identification of a possible modifiable adolescent risk factors – that is, peer victimisation - in driving increased substance misuse in sexual minority groups, which have significant implications for targeted public health strategies for these vulnerable individuals.

**Disclosure of Interest:**

None Declared

